# Inhibitory Effect of *Ulmus davidiana* and *Cornus officinalis* Extracts on Osteoporotic Bone Loss In Vitro and In Vivo

**DOI:** 10.3390/medicina58040466

**Published:** 2022-03-23

**Authors:** Jeonghyun Kim, Chang-Gun Lee, Seung-Hee Yun, Seokjin Hwang, Hyoju Jeon, Eunkuk Park, Seon-Yong Jeong

**Affiliations:** 1 Department of Medical Genetics, Ajou University School of Medicine, Suwon 16499, Korea; danbi37kjh@hanmail.net (J.K.); dangsunsang@naver.com (C.-G.L.); yun41101@ajou.ac.kr (S.-H.Y.); tjrwlshh@naver.com (S.H.); wjsgywn0315@ajou.ac.kr (H.J.); 2 Department of Biomedical Sciences, Ajou University Graduate School of Medicine, Suwon 16499, Korea

**Keywords:** *Ulmus davidiana*, *Cornus officinalis*, primary osteoblasts, primary osteoclasts, ovariectomized mice, herbal medicine

## Abstract

*Background and Objectives:* Traditional herbal medicines are becoming more popular as a complementary medication as they have the advantages of being mostly harmless and safe, causing fewer side-effects than conventional medications. Here, we demonstrate the inhibitory effects of the combination of *Ulmus davidiana* (UD) and *Cornus officinalis* (CO) extracts on osteoporotic bone loss. *Materials and Methods*: This study presented osteogenic effects in primary cultured osteoblasts, pre-osteoblastic MC3T3-E1 cell lines, and osteoclastogenic effects in osteoclasts derived from bone marrow monocytes, and finally, protective effects on bone loss in an ovariectomy (OVX)-induced osteoporotic animal model. *Results*: A significant increase in alkaline phosphatase (ALP) activity was observed following treatment with UD and CO mixtures (8:2, 7:3, and 5:5 ratios) and individual UD and CO extracts, with the highest ALP activity being detected for the treatment with UD and CO extracts at a 5:5 ratio. An optimal ratio of UD and CO (UC) extract promoted osteoblast differentiation in both pre-osteoblastic cells and primary osteoblasts by increasing osteoblastic markers such as *Alpl*, *Runx2*, and *Bglap*. However, treatment with the UC extract inhibited osteoclast differentiation with a decreased expression of osteoclastogenesis-related genes, including *Ctsk*, *Acp5*, *Mmp9*, and *Nfatc1*. In addition, UC treatment prevented osteoporotic bone loss in OVX mice and improved impaired skeletal structure parameters. *Conclusions*: This study suggests that combined UD and CO extracts may be a beneficial traditional medicine for the prevention of postmenopausal osteoporosis.

## 1. Introduction

Bone metabolism is orchestrated by the functioning of osteoblasts (development of bone) and osteoclasts (destruction of bone), resulting in the turnover of a healthy skeleton every 10 years [1]. Dysregulated bone homeostasis triggers a decrease in bone volume and a loss of bone mineral density (BMD), resulting in a high incidence of bone metabolic diseases, including osteoporosis [2]. Osteoporosis is a progressive skeletal disease that results in weak and fragile bones with an increasing incidence of bone fracture [3]. Various factors such as environmental agents, genetic ablation, and hormonal defects are involved in the development of osteoporosis [4]. Various pharmacological drugs have been reported to have the ability to manage osteoporosis by preventing the process or causal agent of osteoporosis [5]. However, several pharmaceutical drugs have been restricted in terms of dosage and frequency because of their negative effects following long-term administration [6].

Alternative medications derived from plants have been broadly applied for the improvement of numerous diseases owing to them causing fewer adverse effects [7]. Previous studies have suggested that combinations of oriental herbal plants exhibit synergistic beneficial effects with minimal side effects compared to individual treatments [8,9]. Recent studies have shown that combined extracts promote synergistic neuroprotective [10], anti-osteoporotic [11] and anti-adipogenic effects [12]. Furthermore, combinations of multiple plant extracts displayed beneficial effects against inflammatory responses in an atopic dermatitis mouse model [13].

*Ulmus davidiana* (UD) and *Cornus officinalis* (CO) are well-known commercialized traditional herbal plants used not only as dietary supplements but also as an oriental medication in East Asia [14,15]. Previous studies have demonstrated that UD extract attenuates angiogenesis via endothelial nitric oxide synthase activation and production in endothelial cells, and inhibits inflammatory processes in rats with lipopolysaccharide-induced lung injury [16] and collagen-induced inflammation [17]. The CO extract has demonstrated anti-allergic properties in RBL-2H3 cells, anti-inflammatory properties in RAW 264.7 cells, and antioxidant activity [18]. Although the ameliorative effects of these traditional plants are known, the combined osteogenic effects of UD and CO extracts have not been studied yet.

In the present study, we examined the inhibitory effect of UD and CO extracts in primary cultured osteoblasts, pre-osteoblastic MC3T3-E1, and osteoclasts derived from monocyte-lineage cells and in an ovariectomized (OVX) osteoporotic murine model.

## 2. Materials and Methods

### 2.1. Preparation of Ulmus Davidiana (UD) and Cornus Officinalis (CO) Extract

CO was provided by Yangpyeong Cornus officinalis grower’s society (YangpyeongGyeonggi-do, Korea), and UD extract was provided by DongWooDang Pharmacy Co., Ltd. (Yeongcheon, Korea). CO and UD extracts were prepared with 30% ethanol at 80 °C for 4 h followed by filtration using filter paper, and hardened low-ash (CHMLAB, Terrassa, Barcelona, Spain) according to the standardized extraction methods (National Herbal Medicine Information, https://www.nifds.go.kr/nhmi, accessed on 21 February 2021). The lyophilized filtrate was stored at −20 °C until use.

### 2.2. Cell Culture

A mouse pre-osteoblast MC3T3-E1 cell-line (subclone 4, CRL-2593, ATCC; Manassas, VA, USA) was grown in a culture medium (alpha-modified minimal essential medium (α-MEM), Gibco, Rockville, MD, USA) containing 10% fetal bovine serum (Gibco) and 1% antibiotic-antimycotic reagent (Gibco). Primary osteoblasts were obtained by the collagenase digestion of mouse calvaria [19]. Neonatal (4–6 pups) Institute of Cancer Research (ICR) mouse calvaria were isolated and digested for 2 h at 37 °C using type II collagenase (Sigma-Aldrich, St. Louis, MO, USA). Then, the digestive solution was neutralized with the culture medium and purified with a Falcon^®^ 40 μm cell strainer (CORNING Inc., Corning, NY, USA). Primary monocytes were obtained from the femurs of mice as previously described [20]. Briefly, bone marrow cells isolated from nine-week-old ICR mice were flushed with culture medium and filtered with a Falcon^®^ 40 μm cell strainer. The suspended cells were centrifuged at 300× *g* for 5 min. The supernatant was removed, and the cells were resuspended in α-MEM supplemented with 50 μg/mL of macrophage-colony stimulating factor (M-CSF; PeproTech, Cranbury, NJ, USA). All stable and primary cells were maintained at 37 °C in an incubator in a humidified atmosphere of 5% CO_2_. The protocol used for the isolation of mouse primary osteoblasts and monocytes was approved by the Institutional Animal Care and Use Committee in Ajou University School of Medicine (2016-0062).

### 2.3. Osteoblast and Osteoclast Differentiation

Osteoblast differentiation was induced by culturing the cells in a media containing ascorbic acid (50 μg/mL) and β-glycerophosphate (10 mM) without changing the medium for 3 days. Isolated monocytes were induced by the addition of 50 ng/mL M-CSF (Peprotech, Cranbury, NJ, USA) and receptor activator of nuclear factor kappa-B ligand (50 ng/mL) (RANKL; Peprotech) in culture medium for 5 days. The induction medium was changed once after 3 days of osteoclast differentiation.

### 2.4. Cell Viability

Cells (1 × 10^4^ cells/mL) were grown in a 96-well culture plate until the cells reached a confluence of 80%. Then, the cells were incubated with UC extract for 3 days (primary osteoblasts) or 5 days (primary osteoclasts). Cell viability was assessed by adding 10 μL of D-Plus™ CCK Cell Viability Assay Kit reagent (Donginbiotech, Seoul, Korea) to the wells and incubated for 90 min at 37 °C. The absorbance (450 nm) was determined by an iMark™ Microplate Absorbance Reader (Bio-Rad, Hercules, CA, USA).

### 2.5. Measurement of Osteoblast and Osteoclast Differentiation

To evaluate osteoblast differentiation, alkaline phosphatase (ALP) activity staining were performed. Cells were lysed with 0.5 M Tris-HCl containing 200 mM EDTA, 1% Triton X-100, and 0.9% NaCl and the ALP activity was determined with 1-Step™ p-nitrophenylphosphate (Sigma-Aldrich). For ALP staining, cells were incubated with the 5-bromo-4-chloro-3-indolyl-phosphate/nitro blue tetrazolium solution (BCIP/NBT, substrate for ALP, Sigma-Aldrich) following fixation with 4% paraformaldehyde for 15 min (BIOSESANG, Seongnam, Korea). An evaluation of tartrate-resistant acid phosphatase (TRAP) activity and staining of osteoclast were performed using an Acid-Phosphatase Kit (Sigma-Aldrich). Images of the stained cells were captured by a Leica microscope (Leica Microcystems, Wetzlar, Germany).

### 2.6. Quantitative Reverse Transcriptase Polymerase Chain Reaction (qRT-PCR)

To determine the expression levels of osteoblastogenesis and osteoclastogenesis, the total RNA was isolated using the TRIzol reagent following the manufacturer’s instructions. The purity and concentrations of RNA were measured by a NanoDrop One/OneC Microvolume UV-Vis Spectrophotometer (Thermo Fisher Scientific, Waltham, MA, USA) at an absorbance of 260/280 nm. Complementary DNA was prepared by 1 µm of total RNA using the RevertAid™ H Minus First Strand cDNA Synthesis Kit (Fermentas, Hanover, NH, USA). qRT-PCR was processed by a SYBR Green I qPCR Kit (TaKaRa, Shiga, Japan) and the fluorescence was measured by a CFX Connect™ Real-Time System (Bio-Rad). The specific primers for osteoblast and osteoclast differentiation are listed in Supplementary Table S1. The relative gene expressions were normalized by mouse *Gapdh* for osteoblasts and mouse *Hprt* for osteoclasts, and the fold change was determined using the 2^−ΔΔCt^ method.

### 2.7. Administration of UC Extract to the Ovariectomized (OVX) Mouse Osteoporosis Model

Sham or OVX-Deutschland, denken and yoken (ddY) mice (eight-week-old) were obtained from Shizuoka Laboratory Center, Inc. (Hamamatsu, Japan) and housed in the Laboratory Animal Research Center of Ajou University Medical Center (22–25 °C and 12 h light/12 h dark cycle). The mice were allowed to consume standard food pellets (Harlan Teklad, Madison, WI, USA) and sterilized water ad libitum. During the whole experimental period, mice were daily administered with methyl sulfonyl methane (MSM; 300 mg/kg/day) or different concentrations of UC extract (100 or 200 mg/kg/day, completely dissolved in PBS) by oral gavage for 12 weeks. The study used MSM as a positive control, because MSM is a well-known commercialized functional compound for the treatment of osteoporosis. All animal experiments were approved and processed under the guidelines of the Institutional Animal Care and Use Committee in Ajou University School of Medicine (2016-0062).

### 2.8. Bone-Mineral Density (BMD) and Micro-CT Analysis

To measure the BMD of mouse femoral bones, the animals were anesthetized by an intraperitoneal injection of zolazepam/tiletamine (Zoletil™ 50; Virbac Laboratories, Carros, France). The BMD was analyzed by a bone densitometer of PIXI-mus (GE Lunar, Madison, WI, USA). For micro-CT analysis, the right femurs of mice were removed and incubated into a fixation solution containing electron microscopy-grade paraformaldehyde (4% in PBS) (BIOSESANG, Seongnam, Korea) for 24 h, and the micro-architecture of the femoral bones was determined using a Bruker micro-CT SkyScan 1173 (Kontich, Belgium). The micro-CT environments were as follows: a current of 400 μA, a voltage of 60 kV, and rotation steps of 360°, and 1280 × 1280 readout of a charge-coupled device (CCD) camera at an exposure of 400 ms. Two- and three-dimensional axial images were reconstructed using Bruker micro-CT NRecon software (North Billerica, MA, USA). Transverse micro-CT images were visualized and the following trabecular outcome parameters were calculated from the region of interest (ROI): trabecular bone volume fraction (BV/TV), trabecular number (Tb.N), trabecular separation (Tb.Sp) and trabecular thickness (Tb.Th).

### 2.9. Statistical Analysis

Data are presented as the mean ± standard error of mean (SEM), by using GraphPad Prism 9.0 software (GraphPad Software, San Diego, CA, USA). Statistical significance was evaluated by Student’s *t*-test for comparison between groups, and one-way analysis of variance (ANOVA) with Tukey’s honest significant difference (HSD) post hoc test for multiple comparisons using the professional Statistical Package (SPSS 11.0 for Windows, SPSS Inc., Chicago, IL, USA). The statistical significance was set at a probability value (*p*) under 0.05.

## 3. Results and Discussion

### 3.1. Mixtures of Ulmus Davidiana (UD) and Cornus Officinalis (CO) Showed Synergistic Osteogenic Effect in MC3T3-E1 Cells

**Figure 1:**
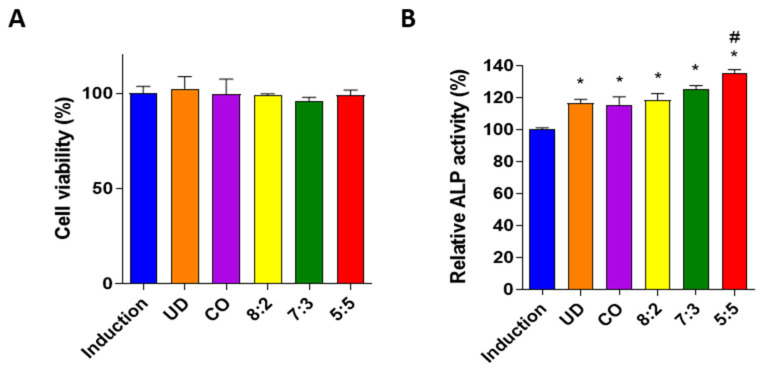
Effects of *Ulmus davidiana* (UD) and *Cornus officinalis* (CO) extract on osteoblast. Pre-osteoblast MC3T3-E1 cells were co-treated with an osteoblast induction medium and 10 μg/mL of UD, CO extract or their combination (8:2, 7:3 and 5:5). After 3 days, (**A**) cell viability and (**B**) ALP activity were measured. The results on the bar-graph are from three independent experiments. UD, *Ulmus davidiana* treatment; CO, *Cornus officinalis* treatment; * *p* < 0.05 vs. induction; ^#^ *p* < 0.05 vs. 8:2 (one-way analysis of variance (ANOVA) with Tukey’s multiple comparison test).

Previous studies have demonstrated that the UD extract prevents bone resorptive effects by decreasing cathepsin K processing in cultured mouse osteoclasts [21], resulting in restored osteoporotic bone loss in ovariectomized (OVX) postmenopausal rats [22]. Additionally, CO has been reported to repress osteoclastogenesis in macrophages derived from bone marrow cells [23] and modulate calcium metabolism and estrogenic regulation [24], indicating the possible mechanisms of CO in the treatment of osteoporosis. These findings implicate the protective effects of both UD and CO extracts on osteoporosis. First, we confirmed the synergistic effects of the combination of UD and CO at different ratios (8:2, 7:3, and 5:5) on osteoblast differentiation, compared to the effect of individual UD and CO extracts in MC3T3-E1 cells. Since UD is a well-known herbal medicine for the treatment of osteoporosis, a higher amount of UD was used in the combined extract. The results of previous studies have suggested the optimal concentrations of the herbal extract [12,25]; hence, a lower concentration of UD and CO mixture (10 μg/mL) was determined for the screening of optimized ratios in this study. Alkaline phosphatase (ALP) activity has been regarded as an indicator of osteoblast differentiation [26]. Therefore, we determined the osteogenic effect of the UD and CO mixture in the pre-osteoblastic cell-line by examining ALP activity. Preosteoblast MC3T3-E1 cells were induced to differentiate by an osteoblast induction medium (β-glycerophosphate (10 mM) and ascorbic acid (50 μg/mL)) and co-incubated with either different ratios of UD and CO mixture or individual UD and CO extracts at 10 μg/mL. As expected, both UD and CO single extracts enhanced ALP activity and did not affect cell viability in the pre-osteoblastic cell line (Figure 1A,B and Supplementary Figure S1). Additionally, the combination of UD and CO at ratios of 8:2, 7:3, and 5:5 at 10 μg/mL significantly increased ALP activity without inducing cytotoxic effects (Figure 1A,B and Supplementary Figure S1). Previous studies demonstrated that the osteogenic effects of the anti-osteoporotic agent could improve osteoblast differentiation by increasing ALP activity in MC3T3-E1 cells [27,28], supporting increased ALP activity in the treatment of UD and CO promoted osteogenic differentiation. The results of additional experiments with UD and CO mixtures and single UD and CO extracts showed that the ALP activity of the optimal UD and CO 5:5 ratio was the highest in MC3T3-E1 cells (Figure 1B). Therefore, the combination of UD and CO extracts at a 5:5 ratio (UC) was used for the subsequent experiments in this study. These results suggest the synergistic effects of the UC extract on the osteogenic effect in MC3T3-E1 cells. However, the understanding of the osteogenic effects of UD and CO is limited to the stable cell-line of pre-osteoblast MC3T3-E1 cells.

### 3.2. UC Increased Osteogenic Effect in Preosteoblast Cells and Primary Mouse Osteoblasts

**Figure 2:**
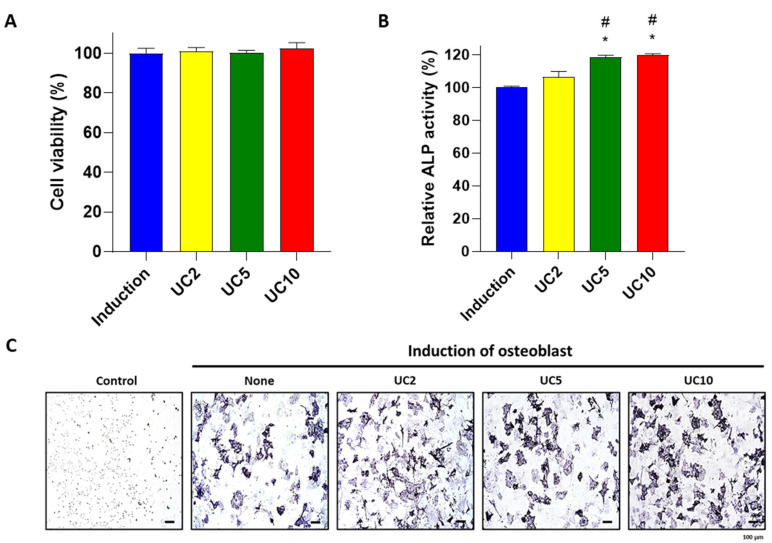
Effects of UC extract on osteoblast activity in primary osteoblasts. Primary osteoblastic cells were co-incubated with an osteoblast induction medium and different concentrations of UC 5:5 extract (2, 5 and 10 μg/mL) for 3 days. (**A**) Cell viability and (**B**) ALP activity were evaluated. (**C**) ALP-stained cell images were obtained by light microscope. The results on the bar-graph are from three independent experiments. UC, *Ulmus davidiana* (UD) and *Cornus officinalis* (CO) 5:5 ratio treatment; * *p* < 0.05 vs. induction; ^#^ *p* < 0.05 vs. UC2 (one-way ANOVA with Tukey’s multiple comparison test).

To further confirm the osteogenic effects of the UC extract on pre-osteoblastic cells, we determined the ALP activity in primary cultured osteoblastic cells derived from mouse calvaria [29]. Primary osteoblastic cells were differentiated by co-treatment with the osteoblast induction medium and several concentrations (2, 5 and 10 μg/mL) of the 5:5 UC mixture. The administration of UC did not influence the viability of the primary cultured cells (Figure 2A). A previous study suggested that the primary cultured system of neonatal mouse calvarial osteoblasts represents a reliable technique for evaluating osteoblasts and the maturation function [29]. Consistent with the results of the MC3T3-E1 cells, UC treatment significantly increased the ALP activity of primary osteoblastic cells (Figure 2B). Furthermore, the number of ALP-stained cells was increased in the UC-treated groups in comparison with the non-treated group (Figure 2C). Consequently, the treatment of UC extract positively promoted osteoblast differentiation in vitro.

### 3.3. UC Increased mRNA Expression of Osteoblastic Makers

**Figure 3:**
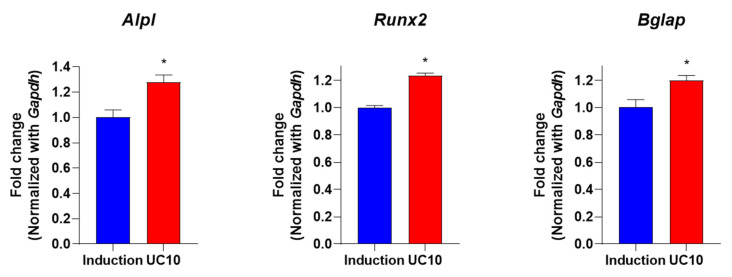
Osteogenic effects of UC extract on osteoblastogensis-related genes in primary osteoblasts. Mouse primary osteoblasts were co-incubated with an osteoblast induction medium and UC 5:5 extract (10 μg/mL). After 3 days, the osteoblastogenesis-related genes such as mouse *Alpl*, *Runx2* and *Bglap* were evaluated by qRT-PCR. The samples were determined by triplicate. UC, *Ulmus davidiana* (UD) and *Cornus officinalis* (CO) 5:5 ratio treatment; * *p* < 0.05 vs. induction (Student’s *t*-test).

Alkaline phosphatase (*Alpl*) is a well-recognized biomarker for the evaluation of osteogenic differentiation in in vitro osteoblastic precursor cells and is associated with increased activity and expression during differentiation [30]. Runt-related transcription factor 2 (*Runx2*) plays a major role in osteoblast proliferation and differentiation [31]. In addition, Bone gamma-carboxyglutamate protein (*Bglap*) is predominantly synthesized by osteoblasts and modulates mineralization during osteogenic maturation [32]. To determine whether the UC extract induces osteoblastogenesis-related markers in primary osteoblasts, mouse primary cultured osteoblasts were co-incubated with 10 μg/mL UC extract and an osteoblast induction medium. After 3 days, the mRNA expression levels of osteoblastic markers were evaluated by quantitative reverse transcriptase PCR (qRT-PCR). A previous study demonstrated that *Alpl*, *Runx2* and *Bglap* are highly expressed during osteogenic differentiation in induced pluripotent stem cells [33]. In the present study, the expression of *Alpl*, *Runx2* and *Bglap* genes were upregulated in the UC treatment group compared to the induction group (Figure 3). These results indicate that the UC extract enhanced osteoblastic differentiation by increasing the expression of osteoblastic markers in primary cultured mouse osteoblasts.

### 3.4. UC Extract Inhibited Differentiation of Osteoclast in Mouse Primary Monocytes

Osteoclasts are family members of macrophages/monocytes and, morphologically, multiple-nuclei giant cells [34]. The abnormal functioning of osteoclast activity is the main cause of skeletal diseases including osteoporosis [35,36]. In addition, many studies have demonstrated that the most frequent therapy for the treatment of osteoporosis is the inhibition of osteoclast activity or differentiation [37,38,39].

To determine the ameliorative effects of UC extract on the osteoclast differentiation, primary monocytes derived from nine-week-old mice were isolated. Osteoclast differentiation is stimulated by the administration of the macrophage colony-stimulating factor (M-CSF) and the receptor activator of nuclear kappa B ligand (RANKL). M-CSF is synthesized by the bone marrow stromal cells and activated pre-osteoclasts as a critical and sufficient factor for osteoclast formation [40]. RANKL is a tumor necrosis factor superfamily and acts as a major stimulator of osteoclastogenesis [41].

**Figure 4:**
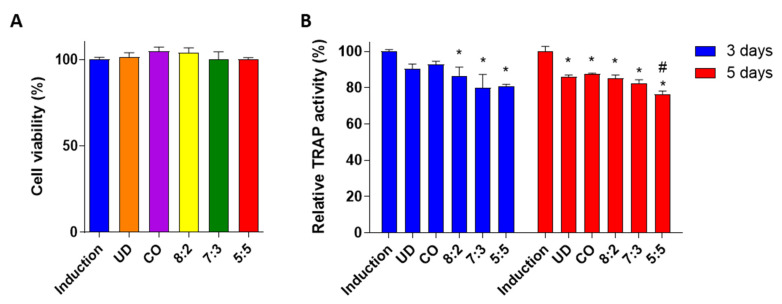
Effects of *Ulmus davidiana* (UD) and *Cornus officinalis* (CO) extract on osteoclast activity. Primary monocytes were co-treated with an osteoclast induction medium and 10 μg/mL of UD, CO extract or their combination (8:2, 7:3 and 5:5). After 5 days of induction, (**A**) cell viability and (**B**) TRAP activity were evaluated. The results on the bar-graph are from three independent experiments. UD, *Ulmus davidiana* treatment; CO, *Cornus officinalis* treatment; * *p* < 0.05 vs. induction; ^#^ *p* < 0.05 vs. 8:2 (one-way ANOVA with Tukey’s multiple comparison test).

In this study, mouse primary monocytes were induced by treatment with an osteoclast induction medium containing M-CSF and RANKL, and co-treatment with either UD or CO extract individually, or different ratios of UD and CO mixture (8:2, 7:3 and 5:5) at 10 μg/mL. During differentiation, osteoclasts express tartrate-resistant acid phosphatase (TRAP) activity to promote migration and bone resorptive activity [42]. All treatments, including individual extracts of UD and CO and UD and CO mixtures (8:2, 7:3 and 5:5) decreased TRAP activity without inducing cytotoxic effects in primary cells (Figure 4A,B and Supplementary Figure S2), indicating an inhibition of osteoclast differentiation. Similar to the results of osteoblast differentiation, the comparison between single and mixture extracts showed that the lowest TRAP activity was observed after treatment with UD and CO 5:5 ratio (UC) (Figure 4B).

**Figure 5:**
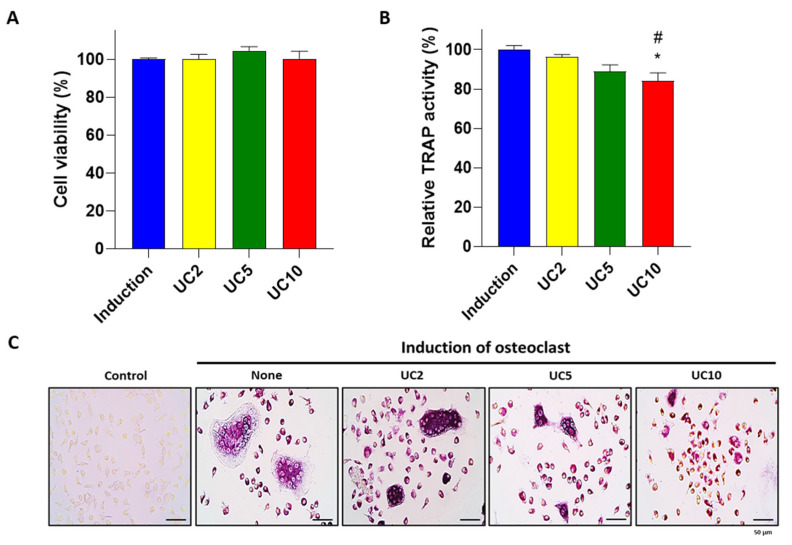
Effects of UC extract on the activity of osteoclasts in mouse primary osteoclasts. Primary monocytes were co-treated with an osteoclast induction medium and different concentrations of UC 5:5 extract (2, 5 and 10 μg/mL). After 5 days of osteoclast induction, (**A**) cell viability and (**B**) TRAP activity were assessed. (**C**) Representative images of TRAP-stained cells were obtained by a light microscope. The results on the bar-graph are from three independent experiments. UC, *Ulmus davidiana* (UD) and *Cornus officinalis* (CO) 5:5 ratio treatment; * *p* < 0.05 vs. induction; ^#^ *p* < 0.05 vs. UC2 (one-way ANOVA with Tukey’s multiple comparison test).

To assess the inhibitory effects of the UC extract on osteoclast differentiation, UC-treated monocytes were assessed by TRAP activity and staining. Consistent with the previous result, UC extract did not alter the proliferation of monocytes (Figure 5A). A previous study suggested that the inhibition of bone destruction is closely related to the reduction in osteoclast activation, formation, survival and progenitor differentiation [40]. In this study, UC extract (10 μg/mL) decreased TRAP activity (Figure 5B) and multinucleated TRAP-positive cell formation (Figure 5C). These results indicate that UC extract inhibited the differentiation and activation of osteoclast by reducing TRAP activity.

### 3.5. UC Decreased mRNA Expression of Osteoclastogenic Makers

**Figure 6:**
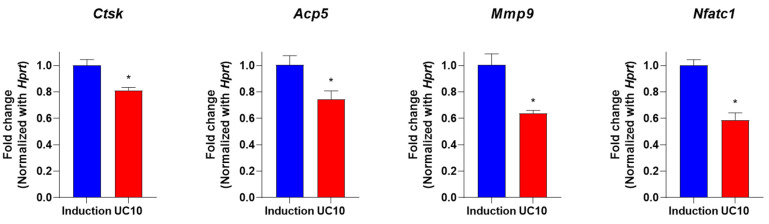
Inhibitory effects of UC extract on osteoclast differentiation-related genes in primary osteoclasts. Mouse primary monocytes were co-incubated with an osteoclast induction medium and UC 5:5 extract (10 μg/mL). After 5 days, the expression levels of mouse *Ctsk*, *Acp5*, *Mmp9* and *Nfatc1* were evaluated by qRT-PCR. The samples were determined in triplicate. UC, *Ulmus davidiana* (UD) and *Cornus officinalis* (CO) 5:5 ratio treatment; * *p* < 0.05 vs. induction (Student’s *t*-test).

The bone resorption process is regulated by various osteoclastogenesis genes, such as cathepsin K (*Ctsk*), tartrate-resistant acid phosphatase (*Acp5,* TRAP) and matrix metalloproteinase 9 (*Mmp9*) [43]. *Ctsk* is a protease which is strongly expressed in osteoclasts and is critical for the degradation of the dominant extracellular matrix (ECM) [44]. *Mmp9* is secreted from osteoclasts as a latent pro-enzyme and is activated and cleaved by *Ctsk*, corresponding to the modulation of the ECM [45]. These genes correlate with the nuclear factor of activated T cells 1 (*Nfatc1*) and act as major regulators of osteoclastic bone resorption [46]. These studies suggested that the expressions of *Ctsk*, *Acp5*, *Mmp9* and *Nfatc1* play a crucial role in determining osteoclast differentiation. Therefore, we determined whether the UC extract could inhibit osteoclastogenesis genes (*Ctsk*, *Acp5*, *Mmp9* and *Nfatc1*) using qRT-PCR. Compared to the induction group, UC extract downregulated the expression levels of *Ctsk*, *Acp5*, *Mmp9* and *Nfatc1* (Figure 6). The results indicate that osteoclast differentiation is inhibited by UC extract via reducing the downregulation of osteoclastic bone-resorptive genes.

### 3.6. UC Extract Decreased OVX-Induced Bone Loss in Osteoporotic Mice

**Figure 7:**
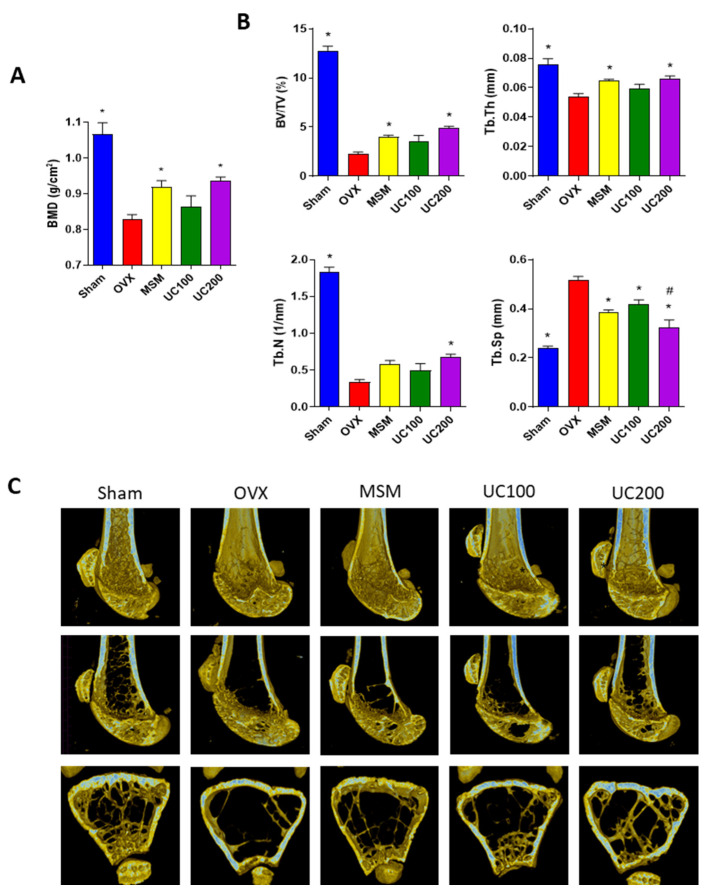
Osteoprotective effects of UC extract on OVX mice. OVX mice (*n* = 5 for each group) were provided with 5:5 ratio of UC extract (100 or 200 mg/kg/day) or methylsulfonylmathane (MSM, 300 mg/kg/day) for 12 weeks. (**A**) Bone mineral density (BMD), (**B**) trabecular parameters such as bone volume (BV/TV), trabecular thickness (Tb.Th), trabecular number (Tb.N) and trabecular space (Tb.Sp) were determined; (**C**) representative images of transverse micro-CT were presented after 12 weeks of administration. UC, *Ulmus davidiana* (UD) and *Cornus officinalis* (CO) 5:5 ratio treatment; * *p* < 0.05 vs. OVX; ^#^ *p* < 0.05 vs. UC100 (one-way ANOVA with Tukey’s multiple comparison test).

Next, we examined the ameliorative effects of the UC extract on osteoporosis a mouse model in vivo. Ovariectomized (OVX) mice are widely used as an animal model for the evaluation of postmenopausal osteoporosis resulting from estrogen deficiency, which leads to BMD loss and a high risk of bone fracture [47]. Most of the drug agents for the treatment of osteoporosis aim to protect bone fractures by attenuating BMD loss, improving bone structural properties and subsequently strengthening the bone [48]. To elucidate whether the UC extract could protect bone loss in OVX mice, OVX mice were daily administered with different contents of UC extract (100 or 200 mg/kg/day) by oral gavage for 12 weeks. Methyl sulfonyl methane (MSM) is a nutraceutical organosulfur compound present in various foods and plants and is a popular dietary supplement for the prevention of osteoporosis [49]. Therefore, this study used MSM (300 mg/kg/day) as the positive control, as previously described [50]. Body weight and food consumption did not differ between the groups without any side effects during the experiment period (data not shown). The measurement of BMD has been widely used for the assessment of anti-resorptive and bone-forming effects of treatments of osteoporosis [48]. In this study, the bone mineral density (BMD) of the right femur was assessed by micro-computed tomography (micro-CT) analysis, and bone morphometric parameters such as bone volume (BV/TV), trabecular thickness (Tb.Th), number (Tb.N) and spacing (Tb.Sp) were analyzed on the last day of animal experimentation. Compared to the sham group, OVX mice presented a reduction in BMD with impaired bone properties (Figure 7). As expected, 12 weeks of MSM administration inhibited the loss of BMD and restored cancellous bone parameters, such as BV/TV, Tb.N, Tb.Th and Tb.Sp. Comparably, UC treatment prevented osteoporotic BMD reduction (Figure 7A) and improved the impaired bone structural properties (Figure 7B). In addition, the UC extract prevented the loss of skeletal structures, as observed in micro-CT images of the transverse and longitudinal sections (Figure 7C). These findings suggest that UC administration inhibits OVX-induced bone loss during the pathogenesis of osteoporosis. Despite the evidence of the beneficial effects of a UC extract in an osteoporotic female mouse model, it is not clear whether the osteogenic effect of UC extract is gender specific. Furthermore, further investigation is required to provide more detail on the protective effects of UC on osteoporotic bone loss.

## 4. Conclusions

In the present study, we tested the osteoprotective effect of a UC extract on the differentiation of osteoblasts and osteoclasts in vitro and on osteoporotic bone loss in OVX mice in vivo. Treatment with the UC extract improved the cellular differentiation of osteoblasts in a MC3T3-E1 mouse pre-osteoblast cell-line and primary osteoblasts by increasing ALP activity and the levels of osteoblast-inducible markers. In contrast, the UC extract inhibited osteoclast differentiation by reducing activity of TRAP and mRNA expression levels of osteoclastogenesis-related genes. In the osteoporotic mouse experiment, UC treatment prevented osteoporotic bone loss and structural compartmentalization in the femoral bone of OVX mice. These results indicate that the UC extract may be a viable therapeutic herbal medicine for the prevention of postmenopausal osteoporotic pathogenesis.

## Data Availability

All data are available on request from the corresponding author.

## Supplementary Materials

The following supporting information can be downloaded at: https://www.mdpi.com/article/10.3390/medicina58040466/s1, Figure S1: Effects of single UD, CO extract and their combination on ALP activity in mouse pre-osteoblast MC3T3-L1 cells. Pre-osteoblast MC3T3-E1 cells were co-treated with an osteoblast induction medium and 10 μg/mL of (**A**) single UD, CO extract or (**B**) their combinations (8:2, 7:3 and 5:5). ALP activity was assessed using p-nitrophenylphosphate. UD, UD treatment; CO, CO treatment; * *p* < 0.05 vs. induction; ^#^ *p* < 0.05 vs. 8:2 (one-way ANOVA with Tukey’s HSD post hoc test). Figure S2: Effects of single UD, CO extract and their combination on TRAP activity in mouse primary osteoclasts. Mouse primary monocytes were co-treated with an osteoclast induction medium and 10 μg/mL of UD, CO extract or their combination (8:2, 7:3 and 5:5) for 5 days. TRAP activity was measured using an Acid-Phosphatase Kit. UD, UD treatment; CO, CO treatment; ^*^ *p* < 0.05 vs. induction; ^#^ *p* < 0.05 vs. 8:2 (one-way ANOVA with Tukey’s HSD post hoc test). Table S1: Gene specific primers used in this study.
