# Supplementary material for: Inhibitory Effect of Ulmus davidiana and Cornus officinalis Extracts on Osteoporotic Bone Loss In Vitro and In Vivo
Source: Medicina (Kaunas). 2022 Mar 23;58(4):466. doi: 10.3390/medicina58040466 (PMC9031261; doi:10.3390/medicina58040466)
Supplement: Supplementary file 1 [file medicina-58-00466-s001.zip › medicina-1628721-supplementary.pdf]

Inhibitory Effect of *Ulmus davidiana* and *Cornus officinalis* extracts in vitro and in vivo

Jeonghyun Kim[1,2,†], Chang Gun Lee[1,2,†], Seung Hee Yun[1,2], Seokjin Hwang[1,2], Hyoju Jeon[1,2], Eunkuk Park[1,2,*] and Seon-Yong Jeong[1,2,*]

Supplementary Table S1. Gene specific primers used in this study.

Gene	Primer sequence
Alpl	Forward: 5′-TCC CAC GTT TTC ACA TTC GG-3′<br>Reverse: 5′-CCC GTT ACC ATA TAG GAT AGC C-3′
Runx2	Forward: 5′-TAA AGT GAC AGT GGA CGG TCC C-3′<br>Reverse: 5′-AAT GCG CCC TAA ATC ACT GAG G-3′
Bglap	Forward: 5′-TAG TGA ACA GAC TCC GGC GCT A-3′<br>Reverse: 5′-TGT AGG CGG TCT TCA AGC CAT-3′
Ctsk	Forward: 5′-AAT ACC TCC CTC TCG ATC CTA CA-3′<br>Reverse: 5′-TGG TTC TTG ACT GGA GTA ACG TA-3′
Acp5	Forward: 5′-TGG TAT GTG CTG GCT GGA AAC-3′<br>Reverse: 5′-GCA GAG GCA TAC TTG TAC CG-3′
Mmp9	Forward: 5′-GCA GAG GCA TAC TTG TAC CG-3′<br>Reverse: 5′-TGA TGT TAT GAT GGT CCC ACT TG-3′
Nfatc1	Forward: 5′-GGA GAG TCC GAG AAT CGA GAT-3′<br>Reverse: 5′-TTG CAG CTA GGA AGT ACG TCT-3′
Gapdh	Forward: 5′-AGG TCG GTG TGA ACG GAT TTG-3′<br>Reverse: 5′-TGT AGA CCA TGT AGT TGA GGT CA-3′
Hprt	Forward: 5′-GAG GAG TCC TGT TGA TGT TGC CAG-3′<br>Reverse: 5′-GGC TGG CCT ATA GGC TCA TAG TGC-3′

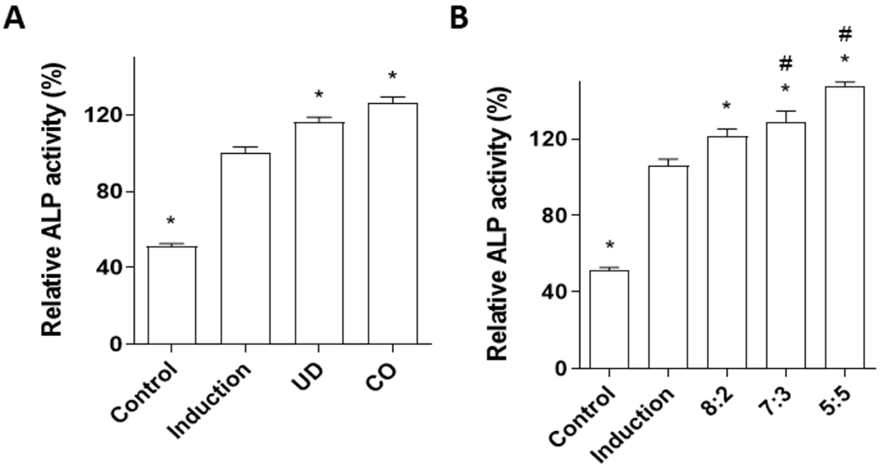

Supplementary Figure S1. Effects of single UD, CO extract and their combination on ALP activity in mouse preosteoblast MC3T3-L1 cells. Preosteoblast MC3T3-E1 cells were co-treated with osteoblast induction medium and 10 μg/mL of (A) single UD, CO extract or (B) their combinations (8:2, 7:3 and 5:5). ALP activity was assessed using p-nitrophenylphosphate. UD; UD treatment, CO; CO treatment, $^{*}p < 0.05$ vs. Induction, $^{\#}p < 0.05$ vs. 8:2 (one-way ANOVA with Tukey's HSD post hoc test).

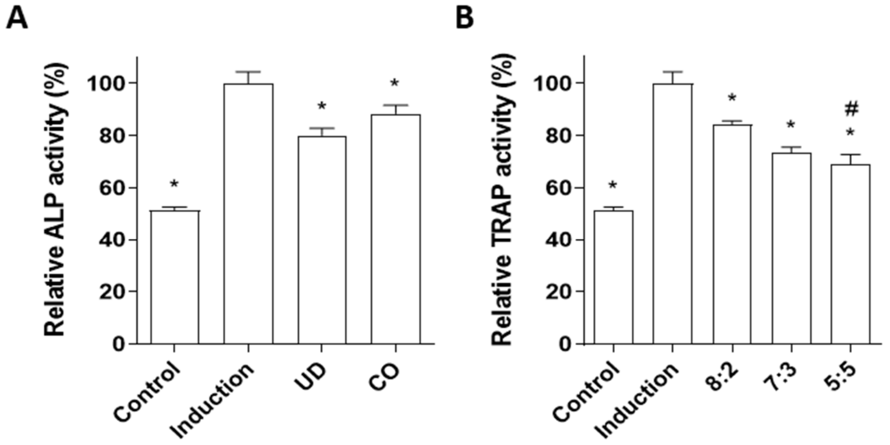

Supplementary Figure S2. Effects of single UD, CO extract and their combination on TRAP activity in mouse primary osteoclasts. Mouse primary monocytes were co-treated with osteoclast induction medium and 10 μg/mL of UD, CO extract or their combination (8:2, 7:3 and 5:5) for 5 days. TRAP activity was measured using Acid-Phosphatase Kit. UD; UD treatment, CO; CO treatment, $^{*}p < 0.05$ vs. Induction, $^{\#}p < 0.05$ vs. 8:2 (one-way ANOVA with Tukey's HSD post hoc test).
